# Linguistic Factors Influencing Speech Audiometric Assessment

**DOI:** 10.1155/2016/7249848

**Published:** 2016-10-18

**Authors:** Martine Coene, Stefanie Krijger, Matthias Meeuws, Geert De Ceulaer, Paul J. Govaerts

**Affiliations:** ^1^Language and Hearing Center Amsterdam, Free University Amsterdam, Amsterdam, Netherlands; ^2^The Eargroup, Antwerp, Belgium; ^3^Department of Otorhinolaryngology, Ghent University, Ghent, Belgium

## Abstract

In speech audiometric testing, hearing performance is typically measured by calculating the number of correct repetitions of a speech stimulus. We investigate to what extent the repetition accuracy of Dutch speech stimuli presented against a background noise is influenced by nonauditory processes. We show that variation in verbal repetition accuracy is partially explained by morpholexical and syntactic features of the target language. Verbs, prepositions, conjunctions, determiners, and pronouns yield significantly lower correct repetitions than nouns, adjectives, or adverbs. The reduced repetition performance for verbs and function words is probably best explained by the similarities in the perceptual nature of verbal morphology and function words in Dutch. For sentences, an overall negative effect of syntactic complexity on speech repetition accuracy was found. The lowest number of correct repetitions was obtained with passive sentences, reflecting the cognitive cost of processing a noncanonical sentence structure. Taken together, these findings may have important implications for the audiological practice. In combination with hearing loss, linguistic complexity may increase the cognitive demands to process sentences in noise, leading to suboptimal functional hearing in day-to-day listening situations. Using test sentences with varying degrees of syntactic complexity may therefore provide useful information to measure functional hearing benefits.

## 1. Introduction

Noise is omnipresent in many aspects of daily life and is known to interfere with oral communication. This is the case in settings as diverse as schools, offices, public transportation, restaurants, and even home. For hearing individuals, understanding speech in such noisy listening conditions can be a serious challenge. In noisy surroundings, auditory perception and processing of speech are even more compromised for individuals with a hearing impairment. Although this so-called “cocktail party effect” has been known for many years [1, 2], it is not yet fully understood how the listener is able to tune in to a single voice in the presence of background noise.

Current models of speech perception take successful speech-in-noise understanding to result from the interaction between auditory, linguistic, and cognitive processing mechanisms: it requires the processing of acoustic signals at the level of the peripheral auditory system to combine with the top-down processing of these input signals at the higher level of the brain by using several components of human cognition, including linguistic knowledge [3, 4].

Recent research regarding functional hearing in daily life situations has investigated speech understanding in noise in several populations, with a special focus on children and elderly listeners with and without hearing impairment. In general, it has been shown that the perceptual accuracy of both children and adults decreases as the signal becomes noisier. The adverse effect of noise has been demonstrated for normal-hearing listeners as well as for listeners with hearing loss. The outcomes of studies targeting child populations indicate that small children require more advantageous signal-to-noise ratios (lower noise levels) than older children and adult listeners [5–7]. A mirror image of the developmental pattern in children is found in elderly adults, a problem to understand speech in noise typically progressing in ageing listeners [4, 8–10].

## 2. Linguistic Cues to Speech Understanding

For both populations, a variety of factors are claimed to contribute to speech-in-noise understanding. Age, noise level, and cognitive performance of the listener all interact with hearing loss. As such, age-related perception difficulties have been related to a decline in cognitive abilities that play a critical role in speech processing in general, including (verbal) working memory and inhibitory control [11–13].

For a few decades already it has been clear that the speech signal itself may also have an important contribution to word and sentence understanding accuracy. When a speech sound in a sentence is deleted and replaced by a noise such as a cough, many listeners are able to restore this portion of missed information. This “auditory induction” ability has been considered a special linguistic application of a general ability to perform phonemic restoration of interrupted signals [14]. Several researchers have also focused on particular features of the linguistic system itself. At the phonosyntactic level, for instance, it has been shown that the articulation of vowels is highly influenced by the consonants by which these vowels are preceded or followed. If for whatever reason listeners have missed part of the incoming message, it may be recovered thanks to information coming from such coarticulation effects [15]. In addition, it has been shown that perception of auditory information is interacting with lexical knowledge as well: when auditory information is ambiguous, listeners have the tendency to make phonetic categorizations that lead to words rather than nonwords (“Ganong effect” [16]), a principle which is the driving force even behind erroneous phonemic replacements in speech audiometric assessment [17].

Some studies have focused more particularly on morphosyntactic features of the linguistic system of the target language [18, 19]. Syntactic complexity and presentation rate of the linguistic message play an important role in sentence understanding, with less accurate performance being associated with syntactically more complex sentences. The effect increases with an increasing speech rate, in the presence of hearing loss and age-related cognitive decline [20]. Further syntactic analysis shows that perception in noise becomes more difficult in sentences with a noncanonical word order and in sentences with complex verb argument structures [21, 22]. Furthermore, speech perception seems to be sensitive to the degree of syntactic complexity of the message. Center-embedded Subject Relative clauses (“the cat that chased the dog meowed”), for instance, yield better sentence repetition scores than sentences with higher syntactic complexity such as center-embedded Object Relative clauses (“the cat that the dog chased meowed”) [20, 23].

## 3. Aim and Research Questions

Building on insights from the literature, it becomes clear that both stimulus- and knowledge-driven processes are highly involved in speech understanding in noise [24, 25]. The exact contribution of auditory and linguistic processes is, however, still under discussion. The present study aims to increase our knowledge with respect to the role of syntactic features in speech-in-noise understanding. More particularly, we investigate how word identification accuracy in noise is influenced by lexical constraints such as the part of speech of the target word, on the one hand, and by syntactic constraints such as the structural complexity of the utterance serving as a natural linguistic context by which this word is surrounded, on the other hand. In addition, we examine the contribution of syntactic structure to the listening effort of the listener by measuring his/her reaction times in responding.

By analyzing verbal repetition scores in adult listeners for words that belong to different parts of speech and that are embedded in utterances with varying syntactic complexity and length, we will be able to better understand an important part of the nonauditory processes involved in speech understanding in noise. In what follows, we will try to answer the following research questions:

1. Is verbal repetition accuracy influenced by the syntactic structure of the carrier sentence, the part of speech of the target word, the length of the carrier sentence, or a combination of these linguistic factors?
2. Is listening effort determined by the syntactic complexity and/or the length of the sentence stimulus?

The answer to these questions will have immediate implications for the audiological practice. Standard speech audiometric test batteries make use of short meaningful sentences that are presented at a given intensity to determine the patient's hearing performance. If sentence materials are used for audiological testing, they need to be balanced in such a way that linguistic complexity contributes as little as possible to the variation in speech audiometric outcomes. If on the other hand no such effect is found, linguistic complexity can be safely “ignored” as a potential confounding factor for this type of audiometric testing.

In the remaining part of the article, we will first try to operationalize the concept of linguistic complexity in a relatively objective way and discuss some psycholinguistic evidence in favor of the role of syntactic and morpholexical cues to speech understanding (Sections 4 and 5). In Section 6 we will present in more detail the development of the test materials and the proposed analyses. Finally in Sections 7 and 8 we will present the results of these analyses and discuss the potential implications for the clinical practice. The general conclusions can be found in Section 9.

## 4. Syntactic Complexity, Cognitive Load, and Speech Understanding

In (psycho)linguistic research, the construct of linguistic complexity has received a good deal of scholarly attention. Although the concept is often used as an index of language proficiency in second language learners, it is generally defined rather sloppily as “the range of forms that surface in language production and the degree of sophistication of such forms” [26]. Similarly, syntactic complexity has been related to the degree of cognitive effort required to produce or interpret particular utterances. Here, syntactic complexity is taken to be “for some reason more difficult, more complex, less entrenched, less frequent, less accessible or in any way cognitively more complex” [27].

More recently, several attempts have been made to operationalize this concept in an objective way [28–31]. Various measures have been proposed, ranging from using pure length (as in the number of syllables, words, or intonation units) of an utterance as a proxy for syntactic complexity to fully fledged in-depth analyses of syntactic tree structures [31]. Although the first approach has the advantage of being readily available, involving no additional structural analysis, it has been shown at many occasions that increased sentence length does not necessarily go hand in hand with increased syntactic complexity. As a matter of fact, the use of utterance length as a measure of linguistic complexity was challenged already a few decades ago, especially by generative approaches to syntax: “it is interesting to note that it is apparently not the length in words of the object that determines the naturalness of the transformation but, rather, in some sense, its complexity. Thus ‘They brought all the leaders of the riot in' seems more natural than ‘They brought the man I saw in'. The latter, though shorter, is more complex” [32, page 477].

Current linguistic research generally agrees upon the fact that utterances with the same number of words or syllables may differ in linguistic complexity resulting from underlying differences in the hierarchical nature of syntactic structure of its constituents. Within a formal framework, the richly articulated internal syntactic structure is captured by using a set of descriptive tools allowing a schematic representation of structural units by means of syntactic trees (see, e.g., [33]). Elementary trees of individual vocabulary items of a language may combine into phrases and sentences; that is, more complex structures are generated by combining syntactic building blocks in well-defined ways, forming so-called simple “canonical” sentences exhibiting the base word order for the particular language in question. Under particular conditions, it is possible to move one or more elements out of their base position into a designated position in the syntactic tree, deriving a sentence with a word order that is different from the canonical one.

Within such a framework, larger syntactic units are represented as nodes in the syntactic tree. Representing linguistic complexity by means of nodes within trees is not merely a formal construct that may be used to describe syntactic variation within a given language in a more systematic way. The way in which the syntactic tree is derived is taken to reflect a psychological entry to syntax, as the different operations underlying syntactic tree formation are representing the functioning of the human parser itself. Current formal syntactic theories are built around a minimalist principle [34] by which syntactic representations should be pure and simple, stripped of all features that are not relevant to the cognitive systems they provide input for. Similarly, syntactic derivations are considered to be subject to principles of economy involving the shortest possible route and the fewest possible steps [35].

Under such a view, linguistically complex structures are cognitively more demanding than their less complex counterparts. One way to quantify syntactic complexity is by counting the number of nodes by which a particular phrase or sentence is dominated, where more nodes indicate a higher degree of formal complexity [29, 36]. The number of syntactic nodes may be taken to reflect part of the computational resources required by the human brain to structure a sequence of words. Roll et al. [31] show that the total number of syntactic nodes in a sentence is a very robust measure of syntactic complexity that is able to account for differences in disfluency of spontaneous speech.

But most often processing difficulties are thought to be proportional to the cognitive cost that comes with syntactic movement [37]. Against this background, it follows rather naturally that utterances which contain a constituent that has been moved out of its base position will show a decreased processing accuracy as compared to utterances with a canonical word order. In order to process a new sentence, the listener needs to activate the syntactic structure; this requires sufficient memory resources. This memory constraint may explain why sentences with a canonical word order are relatively easy to process: for syntactic constituents that appear in their basic sentence-initial position, no memory load is associated with keeping in mind the expectation of them potentially occurring later on in the sentence [36, 38].

Under such a view, passive clauses—that is, structures in which the semantic theme argument (i.e., the participant of a situation upon whom an action is carried out) of the verb occupies the sentence-initial position—will be syntactically more complex than active ones due to a greater cognitive cost for maintaining the possibility of an agent argument (i.e., the participant that carries out the action in a situation) appearing in the clause until encountered after the verb. Compare in this respect (1a-b) from Dutch:

(1) 
  (a) * Die hond bijt de man* (active).
   That dog_AGENT_ bites the man_THEME_.
  (b) * De man wordt gebeten (door die hond)* (passive).
   The man_THEME_ is bitten (by that dog_AGENT_).

Passive clauses such as (1b) are thought to be derived from the underlying canonical sentence of the type Subject_AGENT_ Verb Object_THEME_ by moving the theme into the sentence-initial position, inserting an appropriate auxiliary (Du.* worden*) in front of the past participle and an optional* by*-phrase, the latter referring to the agent of the action expressed by the main verb.

The principle that cognitive costs are proportionally related to the complexity of syntactic movement has been invoked to explain more fine-grained differences in sentence processing between structures that are characterized by movement. As movement has a cognitive cost proportional to the length of the path, longer distance movements are taken to require additional computational resources resulting in reduced interpretation accuracy and longer reaction times [39].

This “shortest-movement” principle has been studied in more detail in the context of relative clauses exhibiting subject-extraction versus object-extraction: in spite of having the same length in words, movement of the relativized noun out of its original subject position of the embedded clause as in (2a) will be shorter and therefore less complex than similar movement out of the object position of the embedded clause (2b):

(2) 
  - * De jongens die [de jongens de oude man kusten], vertrokken gehaast* (subj rel).
  - The boys who the old man kissed, left in a hurry.
  - The boys who kissed the old man, left in a hurry.
  - * De jongens die [de oude man kuste de jongens], vertrokken gehaast* (obj rel).
  - The boys whom the old man kissed, left in a hurry.

If syntactic movement operations are indeed representative for the functioning of the human mind, speech processing of utterances with a longer movement path may be taken to require an additional cognitive effort as compared to utterances characterized by shorter movement. Previous studies have shown that this is indeed the case: when confronted with complex sentences, the comprehension accuracy of hearing impaired listeners drops significantly, due to the fact that extra cognitive load that comes with processing such complex speech is leaving insufficient resources for the comprehension process [40–42]. More recently, Wendt et al. [43] have investigated the extent to which linguistic complexity influences the duration of speech processing in combination with hearing impairment and/or noisy listening conditions in a more objective way by using an eye-tracking approach. More particularly, for participants with hearing impairment, longer processing durations were found for sentence structures with a higher level of linguistic complexity. In noise conditions, the processing durations for complex sentences were linked to cognitive factors such as working memory capacity.

## 5. The Role of Open versus Closed Word Classes in Sentence Understanding

In addition to measures related to syntactic structure, morpholexical features of linguistic units have been shown to influence speech understanding. In the literature, evidence is presented that listeners use their (implicit) knowledge regarding differences between word classes to come to sentence understanding. Current linguistic theories generally take grammatical classes of words (e.g., nouns, verbs, prepositions, and pronouns) to fall into two main groups depending on the context-dependent character of their semantic content: (i) words that do not possess meaningful content by themselves but are mainly used to express a grammatical relationship with other words in the sentence are taken to represent a closed class of function words (e.g.,* pronouns* or* prepositions*), whereas (ii) words that have an autosemantic content allowing for independent lexical meanings are members of an open class of lexical words (e.g.,* nouns *or* verbs*).

There is empirical evidence that the open versus closed class distinction has a reflection in sentence understanding. More particularly, the role of open/closed class words in the processing of spoken sentences has been related to differences in sentence-level prosodic structure: whereas open class words mostly contain at least one stressed syllable, closed class words are most often realized by means of a weak syllable [44]. From an acoustic point of view, the distinction between both classes can often be derived from the presence of full versus reduced vowels. In English, such phonological differences between closed and open word classes are robust and consistent [45, 46]. The human mind has been shown to exploit this phonological information when processing speech, especially with respect to identifying lexical unit boundaries in spoken sentences [47].

A number of studies have investigated whether the phonological differences between closed and open class words trigger differences in auditory processing. The results mainly indicate that open class words have a speech perception advantage over closed class words, probably due to the fact that the presence of a full vowel makes the former stand out more prominently in running speech. It has been shown, for instance, that listeners who are asked to detect a portion of a sentence that was replaced by a noise burst will have less difficulties in doing so when the noise replaces an open class word [48]. Yet other studies have come to rather opposite findings showing that the lexical access process is more complex for open class words than for closed class items [49]. Based on syntactic grounds, similar conclusions have been reached arguing that closed class words mainly encode syntactic information and are therefore subject to relatively little contextual variation making them easier to process [50].

In the next section we will describe how a set of test sentences has been generated and coded in such a way that it is possible to investigate the potential contribution of morpholexical and syntactic features to the identification of words in spoken sentences.

## 6. Materials and Method

### 6.1. Materials

We used the* Linguistically Controlled Sentences for Dutch *(LiCoS), a sentence repetition task consisting of 12 lists of 30 Dutch sentences each containing 2 target words. In this task, sentence repetition accuracy is expressed in terms of the number of correctly repeated target words per sentence (0, 1, or 2). All sentences have been generated in such a way that their semantic predictability is low: they contain no fixed expressions, nor do the two keywords within one sentence belong to the same semantic field (e.g.,* de* ***schoenmaker*** *danst niet vaak met zijn* ***verloofde***, “the shoemaker does not dance often with his fiancée”). Lexical frequency was controlled for by selecting the key words out of the 5000 most frequent words of modern spoken Dutch. Taken together, the 360 test sentences are a representative set of the phonological, lexical, and grammatical variation found in modern spoken Dutch. Half of the test materials have been recorded by a male speaker of Dutch, the other half by a female speaker carefully balancing for the speaker's gender over the different types of sentences.

The linguistic parameters taken into account involve (i) the syntactic structure of the sentences (*SynStr)*, identifying different types of sentences with varying levels of syntactic complexity; (ii) the* part of speech* of the first and second target word in each sentence (*PoS1, PoS2)*, representing 2 major word classes (in agreement with current linguistic approaches, these word classes are representing both open and closed class parts of speech (open: nouns, verbs, adjectives, and adverbs; closed: pronouns, prepositions, determiners, and conjunctions)); (iii) the* length of the sentence* (*SentLen*) expressed in terms of the total number of syllables of the verbal stimulus.

The complete test set may be considered to be a representative sample of the variation of linguistic complexity taking the* Corpus of Modern Spoken Dutch *(Corpus Gesproken Nederlands [51]) as a reference. It therefore contains syntactically “simple” main clauses next to clauses with “medium” complexity (e.g., Passives) and “fully complex” structures (e.g., subject and Object Relative clauses). Variation with respect to the length of the sentence within one syntactic type is limited to 2 syllables per sentence. In a similar vein, all sentence lists were balanced with respect to the length of the key words, each list having a representative proportion of mono-, bi-, tri-, and quadrisyllabic words.

For this study, we have selected a subset of sentences with a length of 11 to 12 syllables equally divided over 6 syntactic types of different complexity. An overview of the syntactic types of the test sentences and of the part of speech of the target words with relevant examples is given in Tables 1 and 2, respectively.

### 6.2. Method

All sentences were presented in a stationary speech noise of −5 dB SNR with the speech noise component fixed at 65 dB SPL. Speech noise was created by spectrally shaping white noise to match the long-term average spectrum of the complete set of sentences. Finally, processing speed was measured in terms of the reaction time of the listener in repeating each individual sentence.

The speech repetition task was performed in one of the quiet rooms of the MediaLab at Free University Amsterdam. In agreement with the local ethical procedures (Ethical Approval EC02.14b), all participants were given oral and written information regarding the goals and procedure of the test and gave their written consent to participate in this study. Prior to the sentence repetition task, the hearing performance of all participants was tested through pure-tone audiometry (500 Hz, 1000 Hz, 2000 Hz, and 4000 Hz). Only participants with hearing thresholds <30 dBHL at all tested frequencies were included. They were given the instruction to repeat as much as they could of each sentence. No additional information was given with respect to which words in the sentence served as target words to measure verbal repetition accuracy.

**Figure 1:**
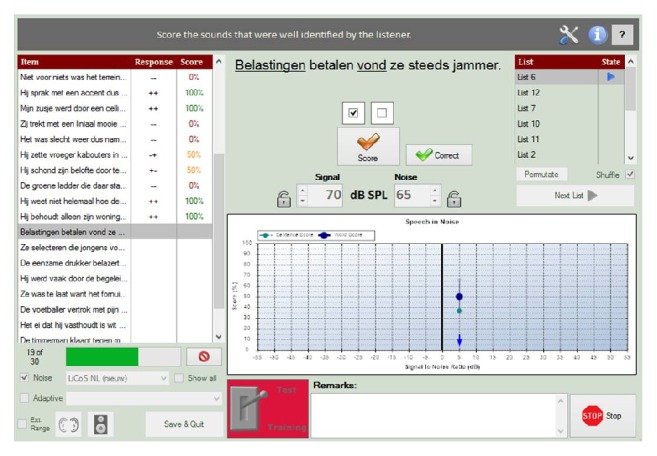
Screen shot of the LiCoS sentence repetition task implemented in A§E 2012 software while presenting the test sentence* belastingen betalen vond ze steeds jammer*, “she always disliked paying taxes.” Sentence repetition scoring is based on the two target words *belastingen* and *vond* (underlined) and may each be flagged when repeated correctly.

The sentences were inserted in A§E 2012® audiometric assessment software [52] and presented in free field with the loudspeakers set at 1-meter distance of the listener. All correct and erroneous repetitions of target words were scored directly in the A§E 2012 software program (Figure 1) by the test administrator, a native speaker of Dutch coming from the same (dialectal) region of the participants. Within sentences, target words were only flagged as being correctly repeated if all phonemes were fully pronounced, including grammatical morphemes such as person and tense or plural markers combining with the nominal or verbal item; for example, repetition of the sentence* de ananas wil* ***de*** *ze niet opeten*, “she didn't want to eat the pineapple” (target words underlined), as* de ananas wil ze niet opeten*, “she doesn't want to eat the pineapple,” yielded a score of 1. In modern spoken Dutch, standard pronunciation often involves the deletion of word final* -n*, regardless of the morphological status of the syllable and lexical category to which it belongs (*mole(n)*, “mill,”* goude(n)*,* “*golden,*”* and* tege(n)*, “against”). Therefore, in nouns with* -en* plural marking, the omission of the entire plural morpheme has been scored as incorrect (*belasting* instead of* belastingen*) whereas the omission of the* mere*  -*n* ending has been flagged as correct (*belastinge *instead of* belastingen*).

### 6.3. Participants

30 normal-hearing Dutch speaking adults were included in this study, involving 8 males and 22 females within the age range of 19–57 years (average age in years = 27.2; SD = 9.89). None of the participants had any experience with speech audiometric testing or sentence repetition tasks. Audiometric data are given in Table 3.

## 7. Results

### 7.1. Syntactic Structure and Sentence Length

**Figure 2:**
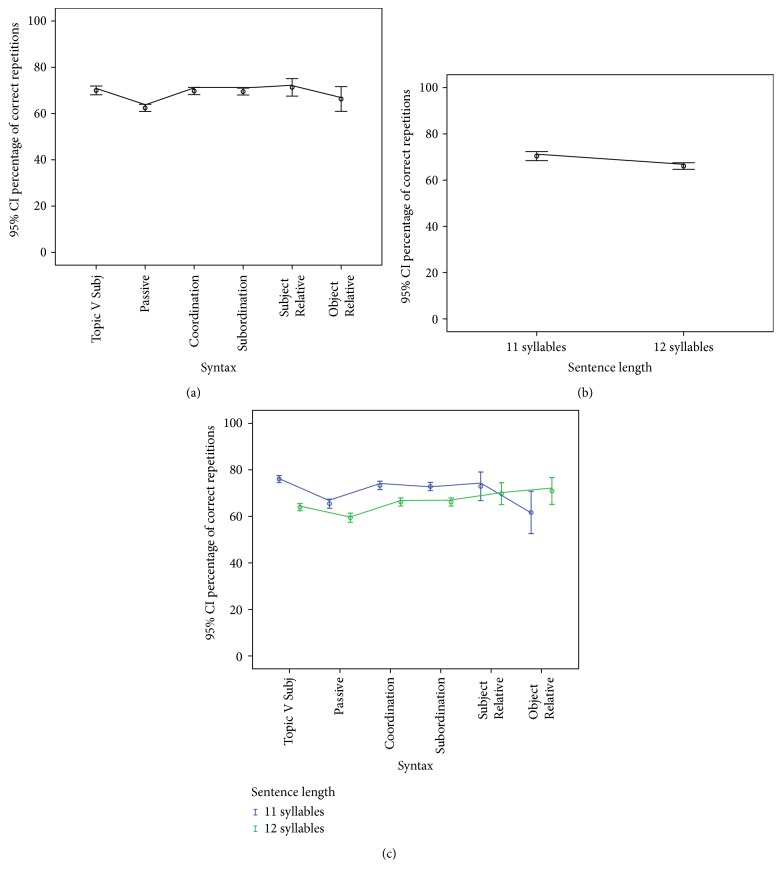
Mean percentage of correct verbal repetition scores with 95% confidence intervals. (a) Comparison based on syntactic structure. (b) Comparison based on sentence length in syllables. (c) Comparison based on the interaction between syntactic structure and sentence length in syllables.

Table 4 presents the means with standard deviations of the percentage of correct repetitions per sentence based on 30 listeners. A repeated measures ANOVA was run using the syntactic structure and the length of the test sentences as within-subjects variables. The results show a significant main effect for both linguistic variables. Firstly, the proportion of correct repetitions revealed to be significantly affected by the syntactic structure of the carrier sentence, *F*(5,67.97) = 4.92, *p* < .007, and *η* ^2^ = .145, representing a small effect. A post hoc analysis showed that listeners obtain significantly lower repetition scores with Passives compared to Topic V Subj structures (*F*(1,29) = 26.15, *p* < .001). Taking Topic V Subj structures as a baseline, all other comparisons between syntactic structures were not significant.  Figure 2(a) depicts the 95% CI of the repetition scores for the different syntactic structures under analysis.

Secondly, a significant main effect of sentence length was also found, with lower correct verbal repetitions for sentences of 12 syllables of length as compared to sentences of 11 syllables, *F*(1,29) = 11.49, *p* < .002, and *η* ^2^ = .284, representing a medium effect (see Figure 2(b)).

Finally, the effect of the interaction between both linguistic variables was tested. The results of this analysis indicate that sentence length is interacting significantly with the syntactic structure of the sentence used as a verbal stimulus (*F*(4,116) = 6.24, *p* < .002, and *η* ^2^ = .177). As can be observed in Figure 2(c), the number of correct repetitions decreases with the increasing length of the sentence, except for Object Relatives. A post hoc analysis of within-subject contrasts for the interaction between syntactic structure and sentence length showed significant effects for all syntactic types (*F*(1,29), Passives = 5.96, *p* = .021; Subordinates = 5.40, *p* = .027; Coordinates = 4.57, *p* = .041; Subject Relatives = 6.65, *p* = .015; Object Relatives = 14.77, *p* = .001).

### 7.2. Part of Speech and Sentence Length

Table 5 presents the means with standard deviation of the percentage of correct repetitions per key word based on 30 listeners for open versus closed word classes.

First, a repeated measures ANOVA was run using the open/closed word class distinction and the length of the test sentences as within-subjects variables. The results show that the percentage of correct verbal repetitions is significantly affected by the type of part of speech (open/closed) of the key words, *F*(1,29) = 4.55, *p* = .042, and *η* ^2^ = .136, and by the length of the sentence, *F*(1,29) = 7.6, *p* < .01, and *η* ^2^ = .208, as well as by the interaction between both of the variables, *F*(1,29) = 19.8, *p* = .001, and *η* ^2^ = .406.

**Figure 3:**
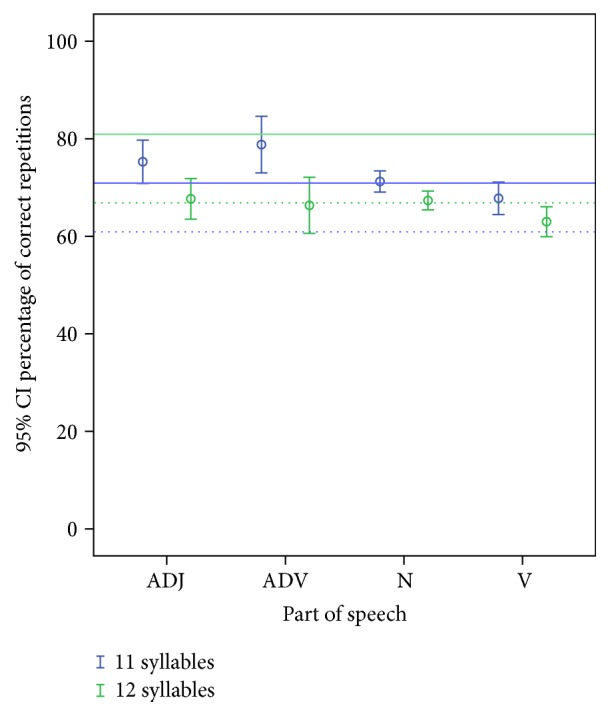
Mean percentage of correct verbal repetition scores with 95% confidence intervals. Comparison based on the part of speech of the key words for lexical categories. ADJ = adjectives, ADV = adverbs, N = nouns, and V = verbs. The dotted and full horizontal lines represent, respectively, the lower and upper bound of the 95% CI for function words (blue lines = 11 syllables; green lines = 12 syllables).

As can be read from the descriptive statistics in Table 5 and Figure 3, in sentences that are 11 syllables long, higher repetition scores are obtained for adjectives, adverbs, and nouns than for function words. In sentences that are 12 syllables long, a reverse effect occurs, the percentage of correct repetitions for adjectives, adverbs, and nouns being situated within the lower bound of the 95% CI for function words. For the category of verbs, however, the number of correct repetitions is low, regardless of the length of the sentences in which they occur.

### 7.3. Ease of Listening

**Figure 4:**
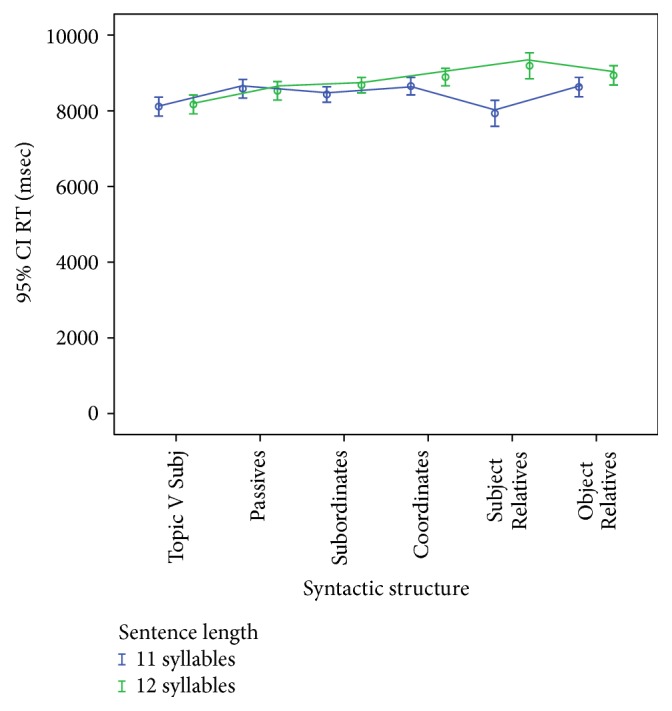
Mean reaction times in repeating the test sentences in milliseconds with 95% confidence intervals. Comparison based on the interaction between syntactic structure and sentence length in syllables.

Table 6 presents the means with standard deviations of the reaction times in milliseconds in repeating each sentence type based on 30 listeners. A repeated measures ANOVA was run using the syntactic structure and the length of the test sentences as within-subjects variables. The results show that both syntax and length have a significant main effect on the reaction times of the listeners (*F*(5,102) = 2.67, *p* = .043, and *η* ^2^ = .084 for syntax; *F*(1,29) = 10.2, *p* = .003, and *η* ^2^ = .260 for sentence length) and that there is a significant interaction effect between both linguistic variables (*F*(5,145) = 3.49, *p* = .005, and *η* ^2^ = .108); see Figure 4.

Post hoc analyses of within-subject contrasts show that Topic Verb Subject sentences require significantly lower reaction times as compared to all other syntactic structures (Passives (*F*(1,29) = 7.68, *p* = .01), Subordinates (*F*(1,29) = 5.83, *p* = .022), Coordinates (*F*(1,29) = 15.18, *p* = .001), Subject Relatives (*F*(1,29) = 4.26, *p* = .048), and Object Relatives (*F*(1,29) = 8.46, *p* = .007)). At the interaction level, no effect of sentence length on reaction times was found for Topic V Subj, Passives, Subordinates, Coordinates, and Object Relatives. Only Subject Relatives revealed to be highly sensitive to sentence length, yielding significantly smaller reaction times for test samples that were 11 syllables long (*F*(1,29) = 7254, *p* = .012).

## 8. Discussion

Our results indicate that language grammar at least partially shapes auditory processing: when confronted with speech stimuli with high linguistic complexity such as passive clauses or Object Relatives, many listeners have difficulties in reconstructing meaningful sentences out of the perceived words. This language-driven aspect of auditory processing becomes noticeable at both the level of accuracy and speed of verbal repetition: while the highest percentage of correct repetition scores is obtained with the sentences that have low syntactic complexity within the test set (Topic Verb Subject structures), the structures with highest syntactic complexity (Object Relative clauses) take most time to be processed. Although syntactic structure has a significant effect on speech perception by its own, it becomes even more pronounced in combination with an increased sentence length. This interaction effect is remarkable given that the difference in length exhibited by the set of test sentences is just 1 syllable. The fact that longer sentences yield significantly lower repetition scores in case of Passives as compared to Topic Verb Subject sentences may be related to the cognitive cost associated with the increased length of the movement path associated with the former structure.

However, our analysis did not reveal any perceptual disadvantage for Subject Relative clauses. If it is indeed the case that the human mind prefers syntactic structures that involve shorter movement over structures with longer—and therefore cognitively more costly—movement paths, this finding is rather unexpected. Relative clauses being generally considered one of the most complex structures in natural language, one would expect them to be associated with a very low repetition accuracy. Yet relative clauses also differ from the other syntactic structures under investigation in that they are typically F(ocus)-marked constructions of which the relativized head noun is standing out in speech (e.g.,* de SOKKEN die ze kwijt was, zijn weer terecht*, “the SOCKS that she had lost have been found again,” versus* ze was haar sokken VERLOREN maar ze zijn weer terecht*, “she had LOST her socks but they have been found again”). According to well-known theories of focus and syntax-prosody interface [53, 54], an F-marked constituent in syntax is interpreted as new or contrastive information in the context. Experimental studies of auditory processing indicate that language users are sensitive to such focused speech materials: not only do words bearing high stress appear to be easier to process during sentence comprehension, they also direct the listener's attention to important elements in the sentence and enable him to make predictions of upcoming accent locations in the entire sentence. These predictions are taken to facilitate sentence understanding [55]. Although our study does not provide direct evidence to claim that focus-marking influences sentence repetition accuracy, the data analyzed here are certainly compatible with current insights regarding the role of multiple components of language in human speech processing. In artificial intelligence approaches to automated speech recognition, for instance, besides expert knowledge regarding the phonetics, phonotactics, and lexicon of spoken language, syntactic and pragmatic features are typically integrated in the design of particular models and algorithms in view of enhancing speech recognition accuracy [56].

To evaluate the single contribution of syntactic structure to speech repetition accuracy, we may compare the two types of relative clauses within our data set. Subject and Object Relative clauses are taken to show similar focus-marking on the head noun; this implies that differences in repetition accuracy may be taken to result from differences in syntactic complexity between the two categories. This is precisely what comes out of the data analysis, Object Relatives exhibiting significantly lower repetition scores than Subject Relatives. Our results are in line with a vast body of literature showing a rather robust asymmetry in the comprehension of Subject versus Object Relatives, the latter being more difficult to understand and involving longer processing times. For the sake of completeness, we would like to point out that, besides syntactic complexity, other factors may influence the accuracy and processing difficulty of different types of relative clauses as well. Animacy, frequency, or internal make-up of the relativized antecedent may influence relative clause understanding, up to the point where Object Relatives will yield better repetition scores than Subject Relatives. In controlled experiments, for instance, it has been demonstrated that placing an inanimate entity in sentential subject position and an animate entity in the Object Relative clause greatly reduces the difficulty normally associated with Object Relative clauses [57].

As for the effect of the different parts of speech of the key words on verbal repetition accuracy, the reduced performance of listeners with verbs as target words is striking. Contrary to adjectives, adverbs, and nouns, repetition accuracy on verbs is as low as that on closed word classes such as prepositions, conjunctions, determiners, and pronouns. We believe that this may be related to the fact that repetitions of verbs have been flagged as correct if and only if they contained the verbal lexeme carrying all its grammatical morphemes (including tense and person/number agreement with the subject). Dutch verbal inflection being characterized by the frequent use of morphemes with low perceptual salience, repetition mistakes often consisted in the omission of such tense or plural markers. Compare in this respect, for instance, the unstressed syllables and nonsyllabic consonants as* -de* marking past tense on verbs like* irriteerde*, “irritated_PAST.SG_,” to the determiner* de*, “the.” In this sense, the perceptual nature of verbal morphemes is not different from that of function words and may therefore explain the observed similarities in repetition performance for both classes of words.

In some cases the omission of these grammatical markers on the verb may even have led to sentences that are well-formed in Dutch (e.g.,* het vest dat ik van je zus* *leen is blauw*, “the jacket that I am borrowing_1.SG.PRES_ from your sister is blue,” instead of* het vest dat ik van je zus leende is blauw*, “the jacket that I borrowed_1.SG.PAST_ from your sister is blue”). The observed similarity in perceptual difficulty between bound and unbound grammatical morphemes is a characteristic that Dutch shares with other Germanic languages such as English. In Romance languages such as Italian, verbal inflections are typically vocalic in nature (e.g., Maria canta_PRES.3.SG._, “Mary sings”) and are therefore expected to trigger less repetition errors. Psycholinguistic research presents experimental evidence in support of the claim that vowels are indeed more perceptually salient than consonants [58]. In atypically developing children, this perceptual factor of morphology has been taken to account for differences in verbal production accuracy: due to the fact that English verb endings are perceptually less salient than their Italian counterparts, English-speaking children with specific language impairment have a harder time acquiring verbal morphology than their Italian-speaking peers [59]. Whether similar perceptual properties of morphemes may be invoked to explain the reduced repetition accuracy of verbs in Dutch speech audiometric testing contexts should be further investigated in a contrastive setting including speech stimuli and native listeners of both types of languages.

Finally, by measuring the reaction times that listeners need to repeat each of the test sentences, we intended to shed some light on the potential differences in listening effort associated with the understanding of sentences with different linguistic complexity. In this respect, our study offers some interesting outcomes: for speech understanding in noise, earlier studies were able to find increased reaction times at particular measuring points during sentence processing indicating an increase in local processing costs triggered by syntactic complexity [21]. Our data show that an effect of syntactic complexity on reaction times also exists at the level of the entire sentence. Prolonged reaction times with relative clauses as compared to Topic Verb Subject sentences may be taken to reflect increased processing times associated with increasing syntactic complexity. Interestingly, longer Object Relative clauses do not need more time to be processed than shorter ones; bearing in mind that they triggered more repetition errors than other syntactic structures, this seems to indicate that whereas pragmatic salience may have a beneficial influence on listening effort, it does not necessarily favor perceptive accuracy.

For the present study, only young hearing participants were recruited. For hearing impaired listeners, and even more so in the case of elderly individuals, the increased reaction times that are associated with understanding syntactically complex sentences such as Object Relatives may be expected to be even more pronounced as more cognitive effort is needed to fill in missing parts of the auditory information leaving less resources to process syntax. A recent study using an eye-tracking paradigm points in this direction: when confronted with linguistically complex sentences, the eye fixations of hearing impaired listeners toward a target picture which matches the acoustically presented sentence are significantly longer than in normal-hearing listeners. Even at high levels of speech intelligibility hearing impaired patients are shown to spend more time processing sentences [43].

Taken together, these findings may have important implications for the clinical practice. Firstly, they illustrate that perceptual accuracy measured in terms of correct verbal repetitions may well represent just one aspect of functional hearing. In spite of good levels of speech intelligibility, the cognitive demands imposed by particular linguistic contexts in combination with hearing loss may lead to suboptimal functional hearing in day-to-day adverse listening situations. In this respect, the duration of sentence processing may reflect the contribution of nonauditory factors to the “ease of language understanding” in the sense of Rönnberg et al. [60].

Secondly, our findings confirm other similar analyses indicating that the choice of test materials used to measure speech perception performance has an important effect on the outcomes [61]. In case speech materials with low linguistic complexity are used, the observed hearing performance accuracy may indicate a considerable benefit obtained from a hearing aid or a cochlear implant while the subjective evaluation by the patient is dissatisfactory [62]. In a recent study [63], it was shown that self-assessment of the ability to perform in particular listening situations significantly correlated with speech perception measured by means of a sentence repetition task while no such correlation was found with phoneme discrimination [63]. If linguistic factors indeed make an important contribution to subjective hearing benefits, the use of test sentences with varying degrees of syntactic complexity may provide useful information with respect to the functional hearing of the patient.

## 9. Conclusion

In current speech audiometric test settings, the hearing performance of patients is typically measured by calculating the number of correct repetitions of a speech stimulus. In this study we have investigated if sentence repetition in noise is influenced by morpholexical constraints such as the part of speech of the target word, on the one hand, and by syntactic constraints such as the structural complexity of the utterance serving as a natural linguistic context by which this word is surrounded, on the other hand. The outcomes of our study showed that variation in verbal repetition accuracy is at least partially influenced by the linguistic make-up of the sentence: at the lexical level, we found that repetition scores are significantly lower with verbs than with nouns, adjectives, or adverbs but similar to prepositions, conjunctions, determiners, and pronouns. The reduced repetition performance for verbs and function words is probably best explained by the similarities in the perceptual nature of verbal morphology and function words in the Dutch language.

At the level of syntax, six categories of structures were compared exhibiting different structural complexity according to the length of the movement path of one or more constituents in the sentence. An overall effect of syntactic structure on speech repetition accuracy was found. The lowest number of correct repetitions was obtained with passive sentences, reflecting the cognitive cost of processing a complex structure in which the semantic object of the verb has been moved out of its base position. The fact that no perceptual disadvantage was found for relative clauses is unexpected but probably best explained by the fact that relativized nouns are generally focus-marked items and are therefore perceptually standing out in the sentence. When such pragmatic factors are controlled for, the negative effect of syntactic complexity becomes noticeable again: worse repetition scores are obtained with syntactically more complex Object Relative clauses as compared to less complex Subject Relative clauses.

Finally, by measuring reaction times in repeating each test sentence, we were able to show that processing speed is dependent upon the same syntactic features. In this respect, similar reaction times with Object Relative clauses as compared to less complex sentence types indicate that whereas pragmatic salience may favor listening effort, perceptive accuracy seems to be mainly determined by syntactic complexity.

Taken together, our findings may have important implications for the audiological practice. Nonauditory factors such as lexical and syntactic features of the target language system may increase the cognitive demands to process sentences in noise. In combination with hearing loss, this may lead to suboptimal functional hearing in day-to-day listening situations even for patients with good speech discrimination outcomes. In this sense, the use of test sentences with varying degrees of syntactic complexity may provide useful information to subjective benefits of particular hearing devices for the patient.

## Figures and Tables

**Table 1:** Inventory of the syntactic types of the test sentences.

SynStr	# syllables	Examples
Topic Verb Subject	11	*Over het algemeen ben jij nogal speels.* Generally (speaking) you are quite playful.
	12	*Tegen de avond zou het kunnen regenen.* By tonight it might be raining.

Passive	11	*Deze acteur wordt door de pers geprezen*. This actor is praised by the press.
	12	*Toetsen worden door de ouders ondertekend.* Written tests shall be signed by the parents

Coordination	11	*Hij gaat naar het zwembad en zij naar de stad.* He is going to the swimming pool and she (is going) into town
	12	*We vonden het erg leuk en bleven dus langer.* We liked it very much and therefore stayed longer

Subordination	11	*Hij dacht niet dat jij die tafel zou kopen.* He didn't think that you would buy that table
	12	*Ze had geluk dat die windhoos haar net mistte.* She was lucky that the tornado just missed her.

Subject Relatives	11	*De meubels die in de schuur staan, mogen weg.* The furniture that is in the barn can be thrown away.
	12	*De schilder die zonet hier was, is nu weg.* The painter who was just here, has left now.

Object Relatives	11	Ze kent geen burger die altijd zijn plicht doet. She doesn't know a citizen who always does his duty.
	12	*De fles die op tafel stond, gooide hij omver.* He threw away the bottle that was on the table.

**Table 2:** Inventory of the part of speech of the target words within the sentences.

Class	PoS	Examples			
Open	Adjective	*Groot*, “great”	*Moeilijk*, “difficult”	*Aanwezig*, “present”	Plaatselijke, “local”
	Adverb	*Al*, “already”	*Even*, “a while”	*Donderdag*, “thursday”	
	Noun	*Vrouw*, “woman”	*Zusje*, “little sister”	*Zakenman*, “businessman”	*Belastingen*, “taxes”
	Verb	*Kent*, “knows”	*Wijzen*, “point”	*Bevallen*, “given birth”	

Closed	Preposition	*Met*, “with”	*Tegen*, “against”		
	Pronoun	*Ze*, “she”	*Ervan*, “thereof”	*Iedereen*, “all”	
	Determiner	*Die*, “that”			

**Table 3:** Participant data.

Participant	Gender	Age	PTA AS	PTA AD
1	Female	56	21.25	17.5
2	Male	22	1.25	5
3	Male	35	8.75	5
4	Female	21	23.75	18.75
5	Female	22	13.75	8.75
6	Female	22	18.75	10
7	Male	21	11.25	10
8	Male	22	13.75	5
9	Female	20	10	6.25
10	Female	19	13.75	10
11	Female	29	10	6.25
12	Female	23	13.75	15
13	Female	19	16.25	13.75
14	Female	22	7.5	5
15	Female	26	7.5	8.75
16	Female	24	27.5	21.25
17	Female	42	27.5	26.25
18	Female	23	12.5	12.5
19	Female	57	17.5	16.25
20	Male	30	12.5	11.25
21	Male	33	28.75	25
22	Male	23	13.75	8.75
23	Female	23	11.25	12.5
24	Female	25	7.5	3.75
25	Female	24	10	10
26	Female	22	10	6.25
27	Female	23	16.25	13.75
28	Female	22	13.75	11.25
29	Male	24	12.5	13.75
30	Female	42	15	15

**Table 4:** Means and standard deviations of the percentage of correct scores for verbal repetition based on syntactic structure and length of the test sentences.

	Mean	SD
*Syntactic structure*		
Topic V Subj	70.04	(11.86)
Passives	62.92	(10.05)
Subordinated	69.51	(8.61)
Coordinated	69.77	(9.25)
Subject Relatives	78.00	(7.32)
Object Relatives	64.13	(8.84)

*Sentence length*		
11 syllables	71.43	(9.49)
12 syllables	65.43	(9.52)

*Syntactic structure ∗ sentence length*		
Topic V Subj		
11 syllables	76.08	(10.06)
12 syllables	64.00	(10.46)
Passives		
11 syllables	65.94	(8.62)
12 syllables	59.90	(10.60)
Subordinated		
11 syllables	72.86	(8.05)
12 syllables	66.17	(7.93)
Coordinated		
11 syllables	73.33	(9.07)
12 syllables	66.20	(8.09)
Subject Relatives		
11 syllables	72.92	(16.44)
12 syllables	69.76	(12.54)
Object Relatives		
11 syllables	61.67	(24.33)
12 syllables	70.95	(15.46)

**Table 5:** Means with standard deviations of the percentage of correct scores for verbal repetition based on the part of speech of the key words and the length of the test sentences.

	Mean	SD
*Part of speech*		
Open	64.08	(7.71)
Closed	69.33	(15.84)

*Sentence length*		
11 syllables	72.28	(14.83)
12 syllables	66.20	(13.45)

*Part of speech* *∗* *sentence length*		
Open		
11 syllables	65.17	(7.51)
12 syllables	63.00	(7.88)
Closed		
11 syllables	64.67	(10.08)
12 syllables	74.00	(19.05)

*Open word classes: part of speech* *∗* *sentence length*		
Adjectives		
11 syllables	75.28	(11.88)
12 syllables	67.67	(5.17)
Adverbs		
11 syllables	78.79	(15.53)
12 syllables	62.99	(8.22)
Nouns		
11 syllables	71.23	(5.80)
12 syllables	67.33	(5.17)
Verbs		
11 syllables	67.78	(8.88)
12 syllables	62.99	(8.22)

**Table 6:** Means with standard deviation of the reaction times in repeating the test sentences in milliseconds based on syntactic structure and length of the test sentences.

	Mean	SD
*Syntactic structure*		
Topic V Subj	8140	(1036)
Passives	8555	(1322)
Subordinated	8555	(975)
Coordinated	8771	(1082)
Subject Relatives	8560	(1638)
Object Relatives	8783	(1426)

*Sentence length*		
11 syllables	8412	(1095)
12 syllables	8665	(1186)

*Syntactic structure* *∗* *sentence length*		
Topic V Subj		
11 syllables	8112	(1148)
12 syllables	8168	(928)
Passives		
11 syllables	8584	(1319)
12 syllables	8527	(1347)
Subordinated		
11 syllables	8432	(878)
12 syllables	8678	(1064)
Coordinated		
11 syllables	8652	(972)
12 syllables	8889	(1187)
Subject Relatives		
11 syllables	7931	(980)
12 syllables	9189	(1919)
Object Relatives		
11 syllables	9629	(1629)
12 syllables	8936	(1197)
